# Microbiota-Induced Epigenetic Alterations in Depressive Disorders Are Targets for Nutritional and Probiotic Therapies

**DOI:** 10.3390/genes14122217

**Published:** 2023-12-14

**Authors:** Shabnam Nohesara, Hamid Mostafavi Abdolmaleky, Jin-Rong Zhou, Sam Thiagalingam

**Affiliations:** 1 Department of Medicine (Biomedical Genetics), Boston University Chobanian & Avedisian School of Medicine, Boston, MA 02118, USA; snohesar@bu.edu; 2 Nutrition/Metabolism Laboratory, Department of Surgery, Beth Israel Deaconess Medical Center, Harvard Medical School, Boson, MA 02215, USA; jrzhou@bidmc.harvard.edu; 3 Department of Pathology & Laboratory Medicine, Boston University Chobanian & Avedisian School of Medicine, Boston, MA 02118, USA

**Keywords:** major depressive disorder, gut microbiome, diet, probiotics, epigenetic modifiers

## Abstract

Major depressive disorder (MDD) is a complex disorder and a leading cause of disability in 280 million people worldwide. Many environmental factors, such as microbes, drugs, and diet, are involved in the pathogenesis of depressive disorders. However, the underlying mechanisms of depression are complex and include the interaction of genetics with epigenetics and the host immune system. Modifications of the gut microbiome and its metabolites influence stress-related responses and social behavior in patients with depressive disorders by modulating the maturation of immune cells and neurogenesis in the brain mediated by epigenetic modifications. Here, we discuss the potential roles of a leaky gut in the development of depressive disorders via changes in gut microbiota-derived metabolites with epigenetic effects. Next, we will deliberate how altering the gut microbiome composition contributes to the development of depressive disorders via epigenetic alterations. In particular, we focus on how microbiota-derived metabolites such as butyrate as an epigenetic modifier, probiotics, maternal diet, polyphenols, drugs (e.g., antipsychotics, antidepressants, and antibiotics), and fecal microbiota transplantation could positively alleviate depressive-like behaviors by modulating the epigenetic landscape. Finally, we will discuss challenges associated with recent therapeutic approaches for depressive disorders via microbiome-related epigenetic shifts, as well as opportunities to tackle such problems.

## 1. Introduction

Depressive disorders are among the most common and complex emotional mental disorders, which annually influence 350 million people worldwide with an average lifetime prevalence of 11–15% [1,2]. Depressive disorders occur across wide ranges of ages from childhood to late life and inflict a high cost on society [2,3]. The prevalence of depressive disorders has doubled or even higher during the COVID-19 pandemic [4]. Depressive disorders are generally characterized by the loss of interest, depressed mood, hopelessness, feelings of guilt or worthlessness, lack of energy, poor concentration, appetite changes, psychomotor retardation or agitation, anxiety, and sleep disturbances causing problems in daily routines, and sometimes are associated with suicidal ideas [5]. When a depressive disorder lasts for a prolonged period of time with moderate or severe intensity, it causes a serious health condition, which is called major depressive disorder (MDD). Recurrence of depressive episodes with stronger severity and less responsiveness to conventional therapeutic approaches has been found among people with MDD, which in turn impacts the quality of life and increases the risk of suicide [6]. 

**Figure 1:**
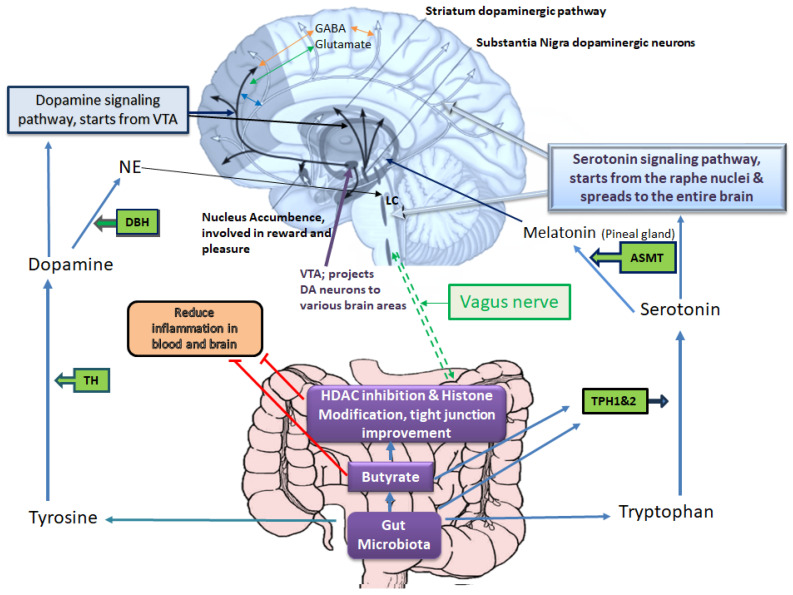
Gut microbiota–host interplays in tryptophan and tyrosine metabolism, which affect mood and emotional states. Based on the monoamine hypothesis of depression, disruptions in brain serotonin, dopamine, or norepinephrine pathways may induce depression. The serotonin signaling pathway starts from the raphe nucleus and spreads to the entire brain, positively influencing the nucleus accumbence responsible for reward and pleasure. The enzymes TPH1 and TPH2 are involved in serotonin synthesis, and TH is involved in dopamine synthesis. Serotonin and dopamine can further proceed to produce melatonin and noradrenaline, respectively. In brain tissue, these neurotransmitters interact with glutamate (excitatory) and GABA (inhibitory) neurotransmitters, which are also implicated in the pathogenesis of depression. The gut microbiota and its metabolites play a key role in providing substrates and influencing enzymes involved in dopamine and serotonin synthesis, consequently affecting noradrenaline (particularly in locus coeruleus, LC) and melatonin (in the pineal gland) production. Melatonin regulates circadian rhythms, which are often disrupted in depression. Additionally, gut microbiota and its metabolites, especially butyrate, help mitigate blood and brain inflammation and influence the activity of the vagus nerve, which directly communicates with the brain in the medulla oblongata. The red T-shape marks indicate inhibition.

Many genetic and environmental factors, such as illicit or prescribed drugs, psychological stress, microbes, and diet, are involved in the pathogenesis of depressive disorders. However, the underlying mechanism of disease pathogenesis is complex and includes the interplay of various key factors related to genetic, epigenetic, and the host immune system, among others. Regarding molecular events that underlie depression, current knowledge greatly supports the monoamine hypothesis of depression [7]. Based on this hypothesis, any perturbation that affects brain serotonin, dopamine, or norepinephrine signaling pathways may induce depression [8,9]. Hence, most of the current antidepressants are designed to increase the level of serotonin (e.g., serotonin-specific reuptake inhibitors), dopamine (e.g., bupropion), noradrenaline (e.g., desipramine), a combination of these neurotransmitters (e.g., serotonin-noradrenaline specific reuptake inhibitors) or inhibit monoamine oxidase enzymes that degrade these neurotransmitters (e.g., monoamine oxidase inhibitors) [10,11,12]. As depicted in Figure 1, several enzymes/genes are involved in processing amino acids to produce these neurotransmitters and several others are involved in their receptors production, their transport or degradation which are targets of current therapeutics [13,14]. For example, serotonin is produced by Tph1 and 2 enzymes in the gut and brain (respectively) from tryptophan, and dopamine is produced from tyrosine (which itself is produced from phenylalanine) by TH (tyrosine hydroxylase) [15,16]. Dopamine then may be processed by DBH (Dopamine β-Hydroxylase) to produce noradrenaline. These neurotransmitters are involved in mood regulation, executive functioning, cognition, motivation, and intellectual function, fundamental aspects of social relationships [17]. MDD also arises from the disruptions of various other neurotransmitter systems, including γ-aminobutyric acid (GABA) and the glutamatergic systems [18,19]. Reduced glutamate levels in specific brain regions correlate with altered emotional responses, offering insights into MDD’s complex neurobiological underpinnings beyond classical neurotransmitters [20].

Among environmental factors, the gut microbiota composition is a key player in serotonin and dopamine production, which may play significant roles in delaying or accelerating depression by regulation of tryptophan availability and, subsequently, serotonin synthesis in the gut and brain cells [21,22]. The gut microbiome–derived metabolites such as butyrate are capable of enhancing the expression and Tph1 activity [23,24,25]. The production of serotonin in the gut, in turn, can influence brain functions via stimulation of the vagus nerve [21], while blood serotonin level affects the blood-brain-barrier (BBB) permeability as well [21]. Moreover, butyrate and epigenetic drugs with similar effects (e.g., sodium valproate) can promote the production of dopamine, noradrenaline, and other related neurotransmitters via enhancing the transcription of the tyrosine hydroxylase (TH) gene [26,27]. 

Other research findings also indicate that modifications of the gut microbiome and its metabolites influence stress-related responses and social behavior in patients with depressive disorders by modulating the maturation of the brain’s immune cells and neurogenesis. In fact, the gut microbiota and its metabolites are involved in a large number of physiological processes like nutrient absorption, strengthening of the intestinal epithelial barrier, facilitating maturation of immune cells, improving functionality of the host immune system, and regulating brain function and human behavior [28,29,30,31]. While the number of microbes in the human gut is several-fold higher than the 30–40 trillion human body cell number, the collective genes of more than 1000 different types of gut microbes are 100–150-fold greater than the human 30,000 genes [32,33]. After the conception of the idea of the microbial–gut–brain axis, it is increasingly becoming clear that this axis is a dynamic, complex, bidirectional communication path that mediates the connection between the gastrointestinal tract (GIT) and the central nervous system (CNS) via several ways including the vagus nerve, neurotransmitters, hormones, microbial metabolites, and the immune system. This axis displays a powerful role in numerous physiological processes like the brain microglia function, the blood–brain barrier integrity and its permeability, and the activity of peripheral immune system cells [34,35]. Accumulating evidence has shown that gut microbiota can be considered as an environment-linked factor that not only shapes the brain via the production of different neurotransmitters but also affects the production of short-chain fatty acids (SCFAs) such as butyrate, acetate, and propionate, which are strong epigenetic modifiers of many genes contributing to the microbiota-gut-brain axis functions. 

Various environmental factors like diet and dietary habits, psychosocial stress, sedentary lifestyle, smoking, antibiotic consumption, chemical substances, and pesticides influence the composition of gut microbiota and the development of depressive disorders [36]. For example, mice with depressive disorders-related behaviors often exhibit altered gut microbiome [37,38]. Furthermore, a growing body of evidence has shown that gut microbiota alterations are associated with the dysregulation of epigenetic mechanisms (e.g., DNA methylation, histone modification, and non-coding RNA-associated gene silencing) and, thereby, the development of depressive disorders [39,40]. Non-coding RNA-associated gene silencing, which is mainly mediated by microRNAs (miRNAs), works between the gut microbiome and intrinsic host factors. For instance, there are significant differences in the expression of miR-294-5p (a miRNA responsible for targeting some key genes in the kynurenine pathway) between germ-free (GF) male mice and conventional mice [41]. Another study by Stilling et al. reported reduced expression of miR-183-5p and miR-182-5p in the amygdala region of the GF mice, and its restoration following microbiome recolonization [42]. In this review, we firstly highlight the potential role of leaky gut, as one of the essential players in development of depressive disorders via epigenetic alterations linked to gut microbiota-derived metabolites changes due to aberrant gut microbiome composition, which finally contribute to the development of depressive disorders. Next, we provide a narrative discussion to introduce potential strategies, such as specific diets and prebiotics or probiotics, that may provide opportunities for the prevention and treatment of depressive disorders. Eventually, we will provide an outlook on the trajectory of recent advancements to present unsolved challenges in emerging therapeutic approaches and how they may be addressed in future research. 

## 2. Roles of Leaky Gut in the Pathophysiology of Depressive Disorders via Alterations of Gut Microbiota-Derived Metabolites Affecting the Epigenetic Landscape 

The intestinal epithelial barrier is composed of a single cell layer that is responsible for the delimitation of the internal milieu from the luminal environment. A major advantage of this restriction is the prevention of entering an expensive range of factors (toxins, pathogens, and antigens) into the lumen, which further results in inflammation, infection, and changes in normal body function [43]. Paracellular transportation across the intestinal epithelium is regulated by tight junctions between adjacent intestinal epithelial cells [44]. The integrity of tight junctions is regulated by endogenous (neural and humoral signals and inflammatory mediators) and exogenous (diet and bacterial metabolites) factors [45]. Some other environmental factors, such as stressful life events, may enhance intestinal permeability and increase the risk of various gastrointestinal disorders and, subsequently, the onset and development of depressive disorders [46,47]. 

It is well-known that gut microbiota is also capable of regulating motility barrier function and visceral perception, and its diversity and composition can by altered by environmental factors, which in turn impact the integrity of tight junctions and hence accelerate (or delay) the development of depressive disorders [48,49,50]. It has been found that an enhanced gastrointestinal permeability with an increased LPS (lipopolysaccharide) translocation from gram-negative bacteria into the blood circulation displays a potent role in the pathophysiology of depressive disorders via activation of the inflammatory response system [46]. In addition to activation of the inflammatory responses, bacterial translocation plays an important role in driving oxidative and nitrosative stress in patients with depressive disorders [51]. The susceptibility of the intestinal barrier is increased secondary to opportunistic microbial inhabitants in a state of microbial dysbiosis. This, in turn, results in the deterioration of the epithelium functions, enhancing localized inflammation, generating inflammatory microbial byproducts, perturbing the functions of other tissues, including the brain, via blood circulation, and increasing the risk of mood-related [52]. Contaminant residues in food products like pesticides are also considered environmental factors with the capacity to disturb gut permeability and enhance inflammation by altering gut microbiome composition [53]. Moreover, it has been reported that psychological stress, such as chronic exposure to limited nesting stress during the first week of the post-natal period, results in enhanced intestinal permeability, reducing fecal microbial diversity, decreasing the abundance of fiber-degrading, butyrate-producing, and mucus-resident microbes as well as increasing the abundance of Gram-positive cocci [54]. The gut microbiota is a key contributor to such effects via their capacity to either enhance or protect against inflammation involving factors affecting epigenetic regulations. For instance, the depletion of bacteria, which produce anti-inflammatory and barrier-strengthening molecules such as butyrate (that enhances the expression of tight junction proteins), results in gut barrier disruption and a loss of protection against epithelial inflammation. *Firmicutes* bacteria of the gut microbiota are also capable of fermentation of carbohydrates to different SCFAs such as butyrate, acetate, and propionate, while the lack of SCFAs (as important and widespread epigenetic modifiers) gives rise to the disruption of intestinal barrier function and secondary inflammation [55]. 

The maintenance of microbial health is largely dependent on the enrichment of microbial SCFA producers and microbial diversity, which are associated with optimal immune function and intestinal barrier integrity. Among SCFAs, butyrate is a master regulator of biological responses of host gastrointestinal health via inhibition of histone deacetylases (HDACs) and binding to specific G protein-coupled receptors (GPCRs) [56]. Butyrate displays a crucial role in numerous physiological processes by crossing across the BBB, activation of the vagus nerve and hypothalamus, and promoting the cholinergic neurons through epigenetic mechanisms [57,58]. Elevated butyrate in the gut contributes to promoting epithelial barrier integrity and inhibiting the host’s systemic inflammation via activation of regulatory T cells [59]. In addition to the activation of regulatory T cells, this gut microbiota metabolite enhances the expression of lncLy6C, which further results in promoting the differentiation of Ly6C^high^ pro-inflammatory monocytes into Ly6C^low/neg^ resident macrophages. This is via binding to the transcription factor C/EBPβ and multiple lysine methyltransferases of H3K4me3 (tri methylation of lysine 4 of the DNA packaging protein Histone H3) and hence improving the enrichment of C/EBPβ and H3K4me3 marks on the promoter region of Nr4A1 gene involved in inflammation regulation and neuroprotection [60]. Moreover, in the CNS, butyrate can enhance the expression of brain-derived neurotrophic factor (BDNF), a main mediator of antidepressant-like effects in animal models, via influencing the hippocampus function [61]. Diet and other environmental factors are considered master regulators of butyrate-producing bacteria and, thereby, the maintenance of intestinal barrier integrity. For example, an omega-3-rich diet is capable of increasing the abundance of butyrate-producing bacteria like *Subdoligranulum* [62]. Collectively, these findings show that butyrate-mediated gut-blood barrier integrity plays a crucial role in mammal physiology and that its disruption may play a role in the pathophysiology of depressive disorders via alterations of gut microbiota-derived metabolites and microbiome-related epigenetic shifts affecting GPCRs and BDNF in the brain, which will be elaborated in the following sections. 

## 3. The Gut Microbiome Composition and Its Role in the Development of Depressive Disorders via Epigenetic Alterations

Several animal studies uncovered that the gut microbiome composition displays a key role in the onset and development of depressive disorders, and altered microbiota profile in the colon has been reported in different animal models of depressive disorders [37,63,64]. Furthermore, significant increases in *Arthromitus* and *Oscillibacter* but decreases in the abundances of several others (e.g., *Lactobacillus*, *Marvinbryantia*, and *Clostridiales incertae sedis*) associated with the deterioration of intestinal barrier function and alterations in the fecal metabolites involved in tryptophan (the precursor of serotonin) metabolism have been found in stress-induced depressed rats [65]. Stress-induced behavioral changes in mice have also been linked to an increase in the abundance of *Odoribacter* and *Alistipes* bacteria correlated with higher blood IL-1α and IFN-γ levels [66]. More evidence of the relationship between depression and altered microbiota composition comes from studies that have shown fecal transplantation of stress-induced depressed mice to normal mice induces depressive phenotypes in the recipient mice due to a decrease in the production of fatty acids (known epigenetic modifiers) that can be treated with a strain of *Lactobacilli* [67]. In another study, while the “relative abundance of Firmicutes, Actinobacteria and Bacteroidetes” was altered in patients with MDD, fecal microbiota transplantation from these patients to germ-free mice induced depressive-like symptoms in the recipient mice but not in germ-free mice receiving fecal microbiota transplantation from normal individuals [68].

In humans, a survey of a large (>1000) cohort followed by validation in independent data sets of >1000 individuals, it was found that the abundance of two butyrate-producing bacteria (*Faecalibacterium* and *Coprococcus*) correlate with better life quality, while *Dialister* and, *Coprococcus* spp. were reduced in depression [69]. In another recent human study on >2500 individuals, depressive symptoms were associated with the abundance of a large number of bacteria (including genera *Hungatella*, *LachnospiraceaeUCG001*, *Lachnoclostridium*, *Eubacterium ventriosum*, *Eggerthella*, *Sellimonas*, *Subdoligranulum*, *Coprococcus*, *Ruminococcaceae*, *Ruminococcusgauvreauii* group, and *Ruminococcaceae* family) which are involved in the synthesis of butyrate, γ amino butyric acid, glutamate and serotonin, key players in depressive disorders [70]. Another clinical study in young adults showed that subjects with MDD exhibited higher levels of specific taxa like *Flavonifractor* and Gammaproteobacteria and lower levels of butyrate-producing, anti-inflammatory bacteria such as *Subdoligranulum* and *Faecalibacterium* [71]. In addition to changes in the abundance of bacteria that produce butyrate (which is involved in histone acetylation, an open chromatin state), alteration of diverse bacterial species may also affect DNA methylation or miRNAs. For example, in a clinical study of obese and non-obese patients with polycystic ovary syndrome (POCS) and higher depression scores vs. controls, while several gut bacteria exhibited significant alterations, this was associated with DNA hypomethylation of the FKBP5 gene, which together with NF-κB mediates inflammation and stress responses, and its increased expression is associated with impaired stress responses and unresponsiveness to antidepressants [72]. Regarding miRNA alterations, the abundances of specific genera such as *Bacteroides* and *Dialister* in MDD patients were highly correlated with the expression of several miRNAs involved in the function of MDD-associated and neurotrophins signaling pathway, circadian rhythm, and dopaminergic synapses among others [73] addressing the involvement of microbiota-miRNA interactions in the microbiota-gut-brain axis. In Table 1, we summarized studies in which differences in the microbial structure and composition via epigenetic alterations confer the development of depressive disorders. 

## 4. Depression and Maternal Diet and Environmental Contaminants Which Affect the Gut Microbiome and Epigenome 

Animal studies have shown that maternal diet during gestation plays an essential role in the health and the neurodevelopment of offspring by modulating the gut microbiome and its metabolites [87]. Unhealthy modern diets such as high-fat diets are capable of inducing maternal dysbiosis, reducing the abundance of butyrate-producing bacteria like *Firmicutes phylum*, associated with an increase in anxiety-/depressive-like behaviors in male and female offspring in mice [88]. Moreover, prolonged high-fat diet feeding could reduce maternal gut SCFAs level, enhancing inflammation, decreasing the abundance of neuroactive metabolites in maternal milk during nursing, and hence increasing anxiety and depressive -like behaviors in both juvenile and adult offspring of obese dams [89]. However, maternal probiotic treatment was capable of increasing gut butyrate and brain lactate in these juvenile and adult offspring, exerting a long-lasting effect on offspring neuroplasticity and their gut–liver–brain metabolome, and thereby promoting resilience to emotional dysfunction induced by maternal obesity [89]. It has been reported that maternal prebiotics (nutrients that influence gut bacterial composition) also affect fecal levels of some bacteria and brain gene expression and behavior in young and adult offspring. For example, maternal Galacto-oligosaccharide prebiotic supplementation in mice could enhance fecal butyrate and propionate levels and reduce anxiety in adult offspring [90]. On the other hand, a maternal low-fiber diet gave rise to impairment of neurocognitive functions and synaptic plasticity in offspring through altering SCFA levels, but butyrate intake could prevent these problems via epigenetic alterations [91]. High-dietary fiber intake could also reduce antenatal obesity-induced postpartum depressive disorders in the maternal mice after the offspring weaning by re-shaping the gut microbiome and increasing the formation of SCFAs (butyrate, acetate, and propionate), and hence suppressing neuroinflammation [92]. 

Early life exposure to environmental chemicals such as pesticides is another common pathogenesis hallmark of depressive disorders that may be associated with the disturbances of gut microbiome structure [93,94]. As an example, low-dose exposure to chlorpyrifos (a common pesticide) during early developmental periods confers perturbations of the gut-brain axis and, thereby, neurobehavioral deficits in offspring [95,96]. Some other pesticides, like glyphosate, by crossing the placental barrier and BBB, exert adverse impacts on neuroplasticity, neurodevelopment, and neuropsychiatric disorders, possibly via changing the gut microbiota and epigenetic programing [97,98,99]. Low-dose exposure to glyphosate during pregnancy and the lactational period also induces anxiety-/depressive-like behaviors and social behavior deficits in female offspring via perturbations of the gut-brain-axis and epigenetic alterations. In an interesting recent study, Buchenauer et al. chronically exposed Balb/cByJ mice (dams) to low doses of glyphosate during pregnancy and the lactational period to examine the effects of maternal glyphosate exposure on the composition of the gut microbiota and the induction of anxiety/depressive-disorders-like behaviors in female offspring [74]. Their results revealed that glyphosate-induced DNA hypermethylation of the TPH2 (involved in serotonin synthesis in the CNS) was associated with its reduced expression in the hippocampus of female offspring and inducing depressive/anxiety-like behaviors as well as social activity deficits. Moreover, changes in the gut microbiota (reduced abundance of *Akkermansia*, butyrate- and propionate-producing bacteria, and elevated abundances of *Alistipes* and *Blautia* bacteria relevant to tryptophan metabolism and depressive disorders) were observed in female offspring after maternal glyphosate exposure. Figure 1. illustrates the connection between gut microbiota products such as butyrate and tryptophan which affect gut and brain serotonin level which in turn is processed to produce melatonin (a key player in circadian rhythm regulation) along with its other functions in mood regulation. 

## 5. Microbiota Derived SCFAs for Depressive Disorders Therapy via Epigenetic Changes

There is a link between SCFAs, cellular metabolism, and transcriptional regulation in the intestine, where microorganisms break down complex fibers and carbohydrates to SCFAs [100,101]. In addition to providing a major energy source for the colon epithelial cells, SCFAs play powerful roles in modulating immune responses and regulating sympathetic nervous system activation by influencing the epigenome and gene expression via inhibiting histone deacetylase enzymes [102,103,104]. More specifically, gut microbiota-derived SCFAs influence histone H3 crotonylation at lysine 18 and brain histone acetylation by controlling the activity of histone deacetylases [105,106,107]. In addition to acting as endogenous HDAC inhibitors, SCFAs influence DNA methylation as well [77,108]. The results of other studies supporting the benefits of SCFAs with similar mechanisms of action in coping with depressive disorders via epigenetic alterations are summarized in Table 2.

## 6. Fecal Microbiota Transplantation and Probiotic Therapy for Depressive Disorders through Epigenetic Changes

Historically, fecal microbiota transplantation (FMT) has been used in Chinese traditional medicine for centuries. However, in modern medical practice, it is more likely that Dr. Ben Eiseman used it for the first time in 1958 to treat four cases of pseudomembranous colitis caused by *Clostridium difficile* infection [120]. By definition, FMT is a simple transfer of fecal matter from a healthy individual into the gastrointestinal tract of a recipient patient [121]. Subsequent studies and clinical trials have further expanded FMT applications in the treatment of various medical conditions related to the gut microbiome and other diseases. Now FMT appears to be a promising strategy for improving human mental health using modulation of the gut–brain axis. However, so far, it has not been considered a treatment option for human diseases until its long-term safety and efficacy are proven [122].

Several animal studies have shown that FMT can be considered a reasonable approach to prevent or treat experimentally induced depressive disorders [123]. It has been reported that FMT from patients with depressive disorders is depressogenic in rats, and FMT from healthy individuals may alleviate depressive-like behaviors [68,124]. Since other studies showed that transferring gut microbiota of depressed human patients to germ-free rats gave rise to depressive-like behaviors in the recipient rats, these findings further support that the gut microbiota may play critical roles in pathways relevant to the pathogenesis of depressive disorders [125]. The evolving depressive behavior upon FMT from depressed human subjects has been attributed to the depletion of *Coproccocus* bacteria, which contributes to butyrate production [124]. It appears that a higher abundance of *Coprococcus* in the fecal microbiota transplanted from healthy individuals may be the mediator of the antidepressant-like behavior in the recipient animals. A more recent study also concluded that an improvement in mental and physical health using FMT is linked to increasing several SCFAs (such as butyrate, 2-methylbutyrate, valerate, and isovalerate) via inhibiting inflammatory responses [126].

Another recent human study revealed that the efficacy of FMT in reducing MDD symptoms in healthy and depressed individuals can be linked to an enhanced abundance of SCFA-producing bacteria such as *Butyrivibrio* and *Faecalibacterium* [127]. As one more interesting example, FMT could improve depressive-like behavior by elevating levels of *Firmicutes*, another butyrate-producing bacteria, reducing the levels of *Bacteroidetes* and *Desulfobacterota* at phylum levels, enhancing the expression of tight junction proteins ZO-1 (TJP1, tight junction protein 1) and OCLN (occludin), preventing the loss of villi and epithelial cells, protecting the mucosal layer function, inhibiting the inflammatory cell infiltration in the ileum, and reducing levels of inflammasomes (NLRP3, ASC, caspase-1, and IL-1β) in rat brain [128]. Zhang et al. reported the involvement of the gut microbiota-circHIPK2-astrocyte axis in depressive disorders and found that FMT from NLRP3 knocked out mice markedly alleviated astrocyte dysfunction and the depressive-like behavior induced by CUSM in recipient mice via inhibition of circHIPK2 expression [35].

Alongside the mounting experimental evidence supporting the efficacy of FMT therapy in numerous diseases, scientists have begun proposing the use of purified and beneficial elements from the gut microbiome as a safer alternative to the potentially risky and uncharacterized FMT. Emerging data now suggest that probiotics might serve vital functions in maintaining intestinal homeostasis by modulating the host’s immune response via the involvement of epigenetic mechanisms [129]. For instance, it is well-known that supplementation of certain microbial strains can be a valid therapeutic approach for depressive disorders by promoting intestinal barrier function, strengthening the epithelium, suppressing oxidative stress, inhibiting neural apoptosis, relieving neuronal cell injury in the hippocampal CA3 regions, and restoring neurotransmitters levels [130,131,132,133]. *Clostridium butyricum* has been found to be a bacterium that secretes a high amount of butyrate, a strong epigenetic modifier and anti-inflammatory agent, and hence has been used as a probiotic for alleviating depressive-like behaviors in a mice model of CUMS [134]. In addition, other butyrate-producing bacteria are considered potential probiotics to relieve depressive-disorders-like symptoms in patients with a disturbed gut microbiome [135].

In another exciting study, Tian et al. used a CUMS mouse model to examine the protective effects of lactic acid bacteria (LAB) treatment on depressive disorders [136]. It was shown that CUMS can be reduced by intervention with specific *bifidobacterium* (*Bifidobacterium breve* (*B. breve*) M2CF22M7 and *Bifidobacterium longum* subsp. *Infantis* (*B. infantis*) E41) in a 5-hydroxytryptophan (an intermediate in serotonin production from tryptophan) dependent and microbiota-regulating manner. As well, B. *infantisE41* could enhance the cecal butyrate level. Tian et al. also explored the protective effects of *B. infantis* strain CCFM687 in a mice model of chronic stress-induced depressive disorders [137]. They found that CCFM687 could increase α diversity and the abundance of butyrate-producing bacteria, which further resulted in (i) an improvement in the stress-induced structural and functional dysbiosis of the gut microbiome, (ii) a reduction in the hyperactivity of the hypothalamic–pituitary–adrenal (HPA) axis response, (iii) inhibition of inflammation, and iv) an increase in the secretion of BDNF in the prefrontal cortex involving the 5-HT1A-CREB-BDNF pathway. Dandekar et al. used a multi-strain probiotic formulation (Cognisol) including *Lactobacillus rhamnosus* UBLR-58, *B. infantis* UBBI-01, *B. breve* UBBr-01, *Bacillus coagulans* Unique IS-2, *Lactobacillus plantarum* UBLP-40, and *Bifidobacterium lactis* UBBLa-70 to prevent anxiety- and depressive-disorders-like behaviors in maternal separation and CUMS models via reshaping the gut microbiome–brain activity [138]. It was shown that antidepressant action of Cognisol is associated with the restoration of acetate, propionate, and butyrate levels in fecal samples; the villi/crypt ratio; the goblet cell count, and increased BDNF and serotonin levels and suppression of inflammation in the hippocampus and/or frontal cortex. Song et al. found that *Roseburia hominis* (*R. hominis*), an obligate gram-positive anaerobic bacterium, is an effective probiotic to alleviate neuroinflammation and depressive behaviors in germ-free rats via suppressing microglial activation, reducing the levels of IL-1α, INF-γ, and MCP-1 in the brain, increasing the serum levels of propionate and butyrate, and subsequently inhibiting histone deacetylases [139]. In addition, in a clinical study, Wang et al. reported potential psychotropic effects of a combined three-strain probiotic intervention (*Pediococcus acidilactici* CCFM6432, *Bifidobacterium longum* CCFM687, and *B. breve* CCFM1025) in human MDD patients and found that not only this combined three-strain probiotic could reduce depressive disorders scores but also improved the patients’ gastrointestinal functions [140].

## 7. Polyphenols and Herbal Medicine as Prebiotics for Depressive Disorders via Epigenetic Changes

Polyphenols and herbal medicine have been known to be good candidates for alleviating depressive-disorders-like behaviors via altering bacterial community structure and distribution [141]. For example, crocetin is one of the most important antidepressant compounds in saffron, which alleviates depressive-disorders-like behaviors by increasing the levels of *Romboutsia* (a bacteria genus that produces butyrate), *Turicibacter*, and *Alistipes* [142]. In a study by Liu et al., they examined the protective effects of Zhi-Zi-Chi decoctions (ZZCD), consisting of Gardeniae Fructus (Chinese herbal name is “zhi zi”) and *Semen sojae praeparatum* (Chinese herbal name is “dan dou chi”), in the treatment of depressive symptoms in a rat model of CUMS [143]. Results of their study supported that ZZCD is capable of inducing an antidepressant effect via re-shaping gut microbiota and facilitating butyrate production, which in turn inhibited the release of pro-inflammatory cytokines and modulated BDNF and neurotransmitters along the gut-brain axis. Another study has shown that Xiaoyaosan also increases intestinal butyrate-producing bacterial diversity and subsequently improves depressive-disorders-like behaviors in rats [144]. Xiong et al. also applied Xiaoyaosan polysaccharide for alleviating CUMS-induced depressive-disorders-like behaviors in rats. In their study, Xiaoyaosan polysaccharide could contribute to reducing CUMS-induced depressive-disorders-like behaviors by increasing the diversity of butyrate-producing bacteria, elevating the abundances of the butyrate-producing bacteria *Roseburia* sp. and *Eubacterium* sp., enhancing the distribution of *Flavonifractor* sp., *Anaerostipes* sp., and *Mediterraneibacter* sp., decreasing the abundance of *Clostridium* sp., and thereby enhancing the content of butyrate in the intestine [145].

Donoso et al. examined the therapeutic potential of different flavonoid and non-flavonoid polyphenols against the depressive- and anxiety-like behaviors induced by maternal separation in rats [146]. They found that maternal separation induces a noticeable reduction in gut microbiota-derived metabolites (acetate, propionate, isobutyrate, and isovalerate), decreases the level of total SCFAs, and branched-chain fatty acids (BCFA), and Xanthohumol treatment could alleviate depressive- and anxiety-like behaviors by restoration of propionate levels and improving isobutyrate and valerate levels. In another study, Yan et al. found that antidepressant effects of a polysaccharide from okra (*Abelmoschus esculentus* (L) Moench) in CUMS-induced mice is associated with inhibiting TLR4/NF-κB pathway and restoring the concentrations of SCFAs like butyric acid, acetic acid, and propionic acid [147]. Additionally, using CUMS in rats, Qu et al. found that some traditional Chinese medicines (TCM) are capable of mitigating CUMS-induced depressive disorders-behaviors by increasing the abundance of *Bacteroidetes* and *Roseburia* (a butyrate-producing bacterium) and reducing the levels of *Firmicutes* and *Ruminococcus* [148]. Tongxieyaofang polysaccharide, a classic TCM prescription (containing Radix Paeoniae Alba, Radix Saposhnikoviae, Rhizoma Atractylodis Macrocephalae, and Pericarpium Citri Reticulatae), could also exert antidepressant and anti-inflammatory activities by increasing the relative abundance of butyric acid-producing bacteria like *Lachnospiraceae bacterium 28–4* and *Ruminococcaceae UCG-014* in a mice model of chronic unpredictable stress [149].

## 8. The Antipsychotic and Antidepressant Drugs also Improve Depression in Part by Modulating Gut Microbiota Associated Epigenetic Changes

Antipsychotic drugs have been known to improve depressive disorders, in part, by restoring levels of butyrate-producing bacteria. In an interesting example, Xu et al. examined the association between gut microbiota composition and depression severity via the characterization of the gut microbiota in depressed BD patients before and after quetiapine administration. Their results revealed that microbial composition changes following quetiapine treatment, and the abundance of different butyrate-producing bacteria (such as genera *Roseburia*, *Faecalibacterium*, and *Coprococcus*) is reduced in untreated patients [150]. Furthermore, psychotropic drugs have been found to exert antidepressant effects by modulating the function and composition of gut microbiota. For instance, commonly used antidepressants, like selective serotonin reuptake inhibitors and tricyclic antidepressants, are capable of influencing microbiome composition, intestinal permeability, and gastrointestinal function [151]. Studies in mice revealed that they recover from the disturbed gut microbial ecosystem and alleviate depressive-like behaviors via epigenetic mechanisms [152].

As another example, Zhang et al. investigated the impact of antidepressants fluoxetine and amitriptyline on gut microbiota composition, diversity, and species abundance in CUMS rats. The protective effects of fluoxetine and amitriptyline against depressive-like behaviors were associated with an increased abundance of butyrate-producing bacterial genera like *Butyricimonas* and other helpful bacteria such as *Parabacteroides* and *Alistipes* [153]. Other drugs like ketamine also have demonstrated abilities for alleviating depressive-like behaviors by elevating levels of butyrate-producing bacteria. In an interesting example, Getachew et al. found that chronic administration of ketamine could exert antidepressant and anti-inflammatory effects by increasing the levels of low-abundance bacteria genera, especially butyrate-producing bacteria like *Lactobacillus*, *Turicibacter*, and *Sarcina* and reducing opportunistic pathogens such as *Ruminococcus* and *Mucispirallum* in male Wistar rats [154].

## 9. Antibiotics for Depressive-like Symptoms by Modulating Gut Microbiota-Related Epigenetic Changes

Owing to unique prebiotic properties in enhancing the growth of beneficial bacteria like *Lactobacilli* and *Bifidobacteria*, antibiotics have attracted much attention from the scientific community for depressive disorders treatment by modulating the gut microbiome composition [155,156]. Three weeks of minocycline treatment, a strong antibiotic, could contribute to alleviating the depressive-like phenotype and reducing inflammation by enhancing the relative abundance of *Clostridiales* Family XIII and *Lachnospiraceae*, families known for their butyrate production [157]. Another study by Yang et al. showed that minocycline treatment for 4 weeks could exert an antidepressant effect in a mice model of CUMS by altering the abundance of specific bacterial species, suppressing neuroinflammation in the hippocampus, and restoring metabolites of gut microbiota such as butyrate level [158]. In a recent study, Rifaximin, a non-absorbable antibiotic with low systemic absorption and high safety, could also ameliorate depressive-like behavior induced by CUMS through modulating microglia function and enhancing the relative abundance of *Ruminococcaceae* and *Lachnospiraceae* that enhance brain butyrate level [159].

## 10. Gut Microbiota-Related Vitamins Modulating Epigenetic Codes in Depressive

Various types of vitamins, such as niacin (vitamin B3), thiamine (vitamin B1), folate, and vitamin K, are produced by the gut microbiome, and their deficiency is associated with several neurological diseases like depression [160]. Gut dysbiosis may impair the synthesis of these vitamins, which in turn may contribute to the pathogenesis of mental diseases. The metabolic synthesis of B vitamins from the gut microbiota is vital for normal brain function [161]. Deficiency in B vitamins is associated with depression development and neuroinflammation [162]. The maternal deficiency of vitamins B6, B9, and B12 has been found to be associated with delayed offspring development and anxiety/depressive-like behaviors via epigenetic alterations [163]. For example, Xu et al. found that deficiencies in B9 and B12 resulted in significantly reduced levels of 5-HT and neuropeptide Y in serum and decreasing levels of BDNF and dimethylated lysine 9 on histone H3 (H3K9me2) in the hippocampus of mice [163]. Neuroprotective effects of niacin against depression are linked to its high-affinity to GPR109A, a G protein-coupled receptor [164]. GPR109A is not only a receptor for niacin but also can act as a receptor for the commensal metabolite butyrate. Activation of GPR109A by niacin or butyrate suppresses colonic inflammation [165]. For example, Wadie et al. reported that the neuroprotective effect of niacin against dextran sulfate sodium-induced depressive-like behavior is mediated via GPR109A-mediated mechanisms [166]. Niacin could also reduce oxidative stress and inflammation and enhance hippocampal levels of occludin, ZO-1, and claudin-5 proteins, suggesting niacin is capable of restoring the BBB integrity [166]. Moreover, it has been found that 14-day treatment with niacin and butyrate significantly influenced vitamin B12, vitamin B1, vitamin B2, and nicotinamide and increased the abundance of butyrate-producing bacteria, including *Firmicutes*, *Lactobacillaceae*, and *Lactobacillus* in weaned piglets [167]. While gut bacteria produce vitamin B12, this vitamin has been known to be a master regulator of the one-carbon metabolism, which contributes to DNA methylation [160]. It has been shown that a single dose of vitamin B12 could alleviate depressive-like behavior in mice after 24 h [168]. On the other hand, chronic vitamin B12 deficiency confers depression-like behaviors in female C57BL/6 mice by changing expression of histone modifying enzymes in brain [169]. In addition, niacin, and vitamin B12 supplementation modulate the diversity of cecal microbiota and increase the abundance of butyrate-producing bacteria. As an example, a significant increase in the abundance of cecal *Faecalibacterium* and a lower abundance of *Acinetobacter* were reported in hens receiving 100 or 400 μg/kg of vitamin B12. Moreover, 400 μg/kg of vitamin B12 in diet could give rise to higher abundance of *Butyricicoccus* and lower abundance of cecal *Bilophila* compared with the control diet [170].

Vitamin D deficiency influences up to 80% of individuals in some countries, and its level is associated with gut dysbiosis and inflammation. It was shown that vitamin D supplementation in healthy individuals increases gut microbial diversity, enhancing the abundance of short-chain fatty acid-producing bacteria, including the *Bacteroidetes* to *Firmicutes* ratio, along with higher abundance of the health-promoting probiotic taxa *Bifidobacterium* and *Akkermansia* [171]. It is interesting to note that vitamin D deficiency gives rise to alterations in the intestinal microbiome and reduces gut B vitamin production, which further elevates inflammation [172]. However, the combination of vitamin D and 100 mg vitamin B complex (except B12) could improve sleep and neurologic symptoms by providing a favorable intestinal environment for returning short-chain fatty acid-producing bacteria like *Firmicutes*, *Bacteroidetes*, *Actinobacteria*, and *Proteobacteria* in humans [172]. Vitamin D deficiency-induced depression-like behavior can also be prevented by folic acid supplements via epigenetic mechanisms. In an interesting example, Tuo et al. found that gestational vitamin D deficiency could induce depression-like behavior in mice offspring by increasing cortical DNA hypomethylation of depression-related genes, but folic acid supplement (which, like vitamin B12, plays a key role in methylation machinery) could alleviate depression-like behaviors in adult offspring via restoring cortical DNA methylation [173]. In addition to above-mentioned vitamins, it has been reported that dietary vitamin K can also act as a protective cognitive factor, and certain microbiota-derived vitamin K isoforms are linked to cognitive functions in older human adults [174,175].

## 11. Potentials and Challenges for Translating Gut Microbiome Research to the Clinic for Treatments of Depressive Disorders

Currently, microbiome-targeted medicines present a large number of unexploited therapeutic opportunities for improving mental health. Translation into commercially successful medicines is possible if research scientists focus on functional aspects of the human microbiome when designing new therapeutic approaches and tackling several challenges [176]. Accumulating evidence demonstrates that diet and environmental exposures like antimicrobial use heavily influence gut microbiome strain content and encoded gene function [177,178]. In order to accelerate clinical translation and enhance our understanding of how gut microorganisms and their bioactive metabolites affect a variety of aspects of human physiology during depression, descriptive microbiome investigations should be linked to mechanistic studies. In fact, although recent studies have focused on associations of microbiome with health or disease, it is hard to create a causal or consequential relationship between a specific microbe and a specific disease. Hence, so far, it has been difficult to denote the precise implication of the candidate microorganism for its beneficial or harmful effects on specific diseases when there is a correlation between a given bacteria and a disease phenotype [179,180].

Another challenge for accelerating the clinical translation of microbiomes for the treatment of depressive disorders is differences in microbiome composition and configuration among various human populations and across different areas around the world and ethnicities, which hamper a better understanding of pathological gut microbiome [181,182,183]. In order to increase our understanding of variation in human microbiome functions and to obtain greater insights into how environmental exposures interrelate with microbiome genomic content and function at different periods of human development and across health gradients, it is necessary to precisely evaluate the human microbiome structure worldwide in different developmental periods. There is an urgent need to obtain this information in standardized and longitudinal human microbiome studies with large sample sizes since features of microbiomes alter strikingly in early life, in advanced age, in different seasons, and in geographic locations across healthy populations.

The translation of animal microbiome findings to humans is another challenge due to major differences in animal and human genome and microbiome configurations [184]. In some cases, human studies fail to replicate animal findings related to using microbiomes for the treatment of depressive disorders. One of the practical solutions for unambiguous determination of microbial contributions to the development or prevention of depressive disorders and other serious human diseases is an investment in the development of new tools, such as in vitro or in vivo model systems, to recapitulate observations in humans and precise examination of microbiome and host transcription and translation within human samples [185]. Moreover, high-throughput screening efforts and advancement in selective gut microorganism enrichment, isolation, and culture would provide an opportunity for researchers in the field to culture a specific microorganism in quantities compatible with prolonged in vivo testing. In addition, to accelerate the translation of the findings of microbiome studies in animals to humans, highly likely non-human primates are better alternatives to mimic the human depressive host–microbiome interaction than rodents [186].

Despite these challenges and the fact that most of the previous reports did not consider lifestyle factors, seasonal variations, disease heterogeneity, and medication effects in their studies [187], current data promise therapeutic opportunities for improving mental health. In addition to prebiotics and probiotics, several studies have shown some antibiotics are capable of alleviating depressive-like behaviors via modulating microbiome-related epigenetic modifications, though some of them may have detrimental effects. For example, in addition to their bactericidal or bacteriostatic functions, traditional antibiotics like fluoro-quinolones and β-lactamics profoundly influence the gut microbiome composition, mainly via reducing useful bacteria (e.g., *Bifidobacteria*) and increasing pathogenic bacteria like *Enterobacteriaceae* [188].

## 12. Conclusions

**Figure 2:**
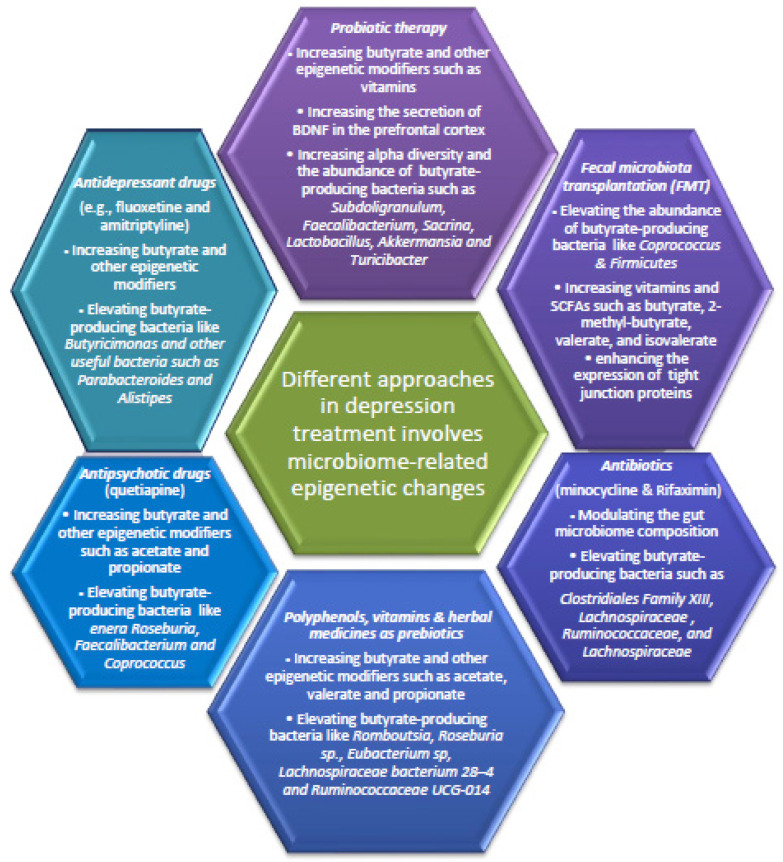
Various approaches in depression treatment involve gut microbiota-related epigenetic modifications. Diet, prebiotics, probiotics, fecal microbiota transplantation (FMT), antibiotics, antipsychotics, and antidepressant drugs may alleviate depression via microbiome-related epigenetic modification involving butyrate and other SCFAs, in particular. These interventions are also capable of increasing the microbiome α diversity, the abundance of butyrate-producing bacteria, the expression of tight junction proteins (ZO-1 and occludin), the secretion of BDNF, the level of butyrate and other epigenetic modifiers that mitigate inflammation.

Currently, the link between microbiome-related epigenetic alterations and depressive disorders has attracted tremendous attention for the treatment of patients with depressive disorders. As shown in Figure 2, even classic therapeutic approaches in the treatment of depression involve microbiota-related epigenetic modifications. A deep understanding of such therapeutic approaches and underlying mechanisms will pave the way for research scientists to prevent or minimize depressive-like behaviors via modulating microbiome-related epigenetic modifications. However, various challenges remain to be considered in this field. A large number of the existing studies possess small samples, and therefore, there is a lack of statistical power to provide robust and reproducible associations. Hence, studies with larger sample sizes and refined experimental design should be implemented to obtain reproducible associations and more precise insights in the field.

It is likely that the current and more advanced upcoming methods of 16S rRNA sequencing will be able to precisely detect pathogenic bacteria involved in depression in the coming years. Then, scientists may design pathogen-specific (like antibiotics) and potentially patient-specific therapeutics for depression and other mental diseases. As an alternative to antibiotic usage against potentially harmful bacteria involved in depression, it is possible to produce vaccines against pathogenic bacteria to eliminate their systemic impacts on the host immune system and curb secondary inflammation, which influences the epigenetic landscape, particularly during critical developmental periods.

## Figures and Tables

**Table 1:** Experimental and clinical evidence linking epigenetic alterations to gut microbiota dysbiosis.

Type of the Study	Key Findings	Epigenetic Alteration	Ref.
Experimental study in mice after maternal glyphosate exposure	DNA hypermethylation of several genes and increased abundance of *Alistipes* and *Blautia* (bacteria involved in tryptophan metabolism) and decreased abundance of *Akkermansia* in offspring after maternal glyphosate exposure	DNA methylation	[74]
Clinical study in obese patients with Polycystic ovary syndrome (POCS)	Association between reduced FKBP5 DNA methylation and stress in obese PCOS patients, particularly with higher depression score; reduced bacterial diversity; higher abundance of *Bacteroides* and *Megamonas*; reduced abundance of *Bacteroidetes* and *Proteobacteria*	DNA methylation	[75]
Clinical study in individuals with low self-esteem, an indicator of depressive	Differences in the immune-epigenetic-gut microbiome axis and DNA methylation at immune-metabolic genes in monocytes and deficiencies in regulatory activity of adiponectin and, thereby, downstream targets of inflammation and gut dysbiosis	DNA methylation	[76]
Clinical study, depressive symptoms in Parkinson’s Disease (PD)	Reducing counts of genera *Roseburia*, *Romboutsia*, and *Prevotella* relevant to depressive symptoms in PD patients and lower fecal butyrate levels association with DNA methylation alteration in leucocytes and neurons	DNA methylation/Histone acetylation	[77]
Experimental study in mice after subchronic and chronic exposure to glyphosate-based herbicide	Decreasing abundance of butyrate-producing bacteria (Firmicutes and Lactobacillus) and other bacteria such as Corynebacterium and Bacteroidetes and, thereby, increasing anxiety and depressive behaviors	Histone acetylation	[78]
Transplanting the gut microbiota from patients with alcohol use disorder to mice	Significant alterations in the microbiome composition and lower hepatic synthesis of β-hydroxybutyrate (BHB) and hence increasing depressive-disorders-like behavior	Histone acetylation	[79]
Experimental study in mice, chronic social defeat	Reducing the frequency of G-protein-coupled receptors owing to significant reductions in the diversity and abundances of numerous bacterial genera involved in the production of butyrate and propionate (e.g., *Akkermansia* spp.) and hence increasing depressive disorders like behaviors	Histone acetylation	[80]
Experimental study, gut microbiota-absent mice	Gut microbiota dysbiosis and association between lysine acetylation alterations and mitochondrial dysfunction in the brain (hippocampus) and identifying 986 lysine acetylation sites in 543 proteins relevant to MDD	Lysine acetylation	[81]
Clinical study, MDD vs. the control group	Significant differences in *Bacteroides* and *Dialister* abundance and expression of six fecal miRNAs (miR-1246, miR-579-3p, miR-1276, miR-1976, miR-3144-3p, miR-4488) in cases vs. controls	microRNAs (miRNAs)	[73]
Clinical study in patients with depressive disorders vs. controls	Significant alterations in the composition of fecal microbiomes Involvement of two microbial-regulated lncRNA–miRNA–mRNA ceRNA regulatory networks in depressive disorders-related neurodevelopment in patients	miRNAs	[82]
Experimental study in mice examining the effects of gut microbiota on miR-206-3p	Increasing degeneration of mitochondria and synapses in the hippocampus and hence enhancing depressive disorders/anxiety-like behaviors by gut microbiota-induced microRNA-206-3p in mouse brain tissues	miRNAs	[83]
Experimental study, LPS-induced depressive-like behavior in mice	Regulating gene expression of Nfatc4 and miR-149 (or miR-7688-5p) in the PFC by gut microbiota and blocking LPS-induced depressive-disorders-like behavior by inhibition of miR-149 using antagomiR-149	miRNAs	[84]
Experimental study in the hippocampus of germ-free mice	Disruption of RNA transcription and post-transcriptional regulation of a lincRNA–miRNA–mRNA network including 12 lincRNAs, six miRNAs, and 47 mRNAs during gut microbiota dysbiosis and hence generating depressive- and anxiety-like behaviors	miRNAs	[85]
Clinical study in Chinese patients with depressive disorders	gut microbiome diversity regulates microRNA expression in the brain and hence influences SERPINA5, a microbe-associated gene, and other spatially close genes involved in accelerating or delaying the development of depressive disorders	microRNA	[86]

**Table 2:** Studies supporting the benefits of SCFAs in coping with depressive disorders.

Experiment	SCFAs Use	Key Findings	Ref
Chronic mild stress or maternal deprivation for the induction of depressive-like behaviors in adult Wistar rat	Sodium butyrate	Reversing the depressive-like behaviors following treatment with sodium butyrate	[109]
Lipopolysaccharide-induced depressive-disorders-like behaviors in mice	Sodium butyrate	Reducing the LPS-induced depressive state via Iba1 hippocampal expression changes and microglia activation by HDAC inhibitors	[110]
Maternal deprivation or chronic mild stress in adult Wistar rat	Sodium butyrate	Reversing mitochondrial alterations in the striatum of rats and depressive-like behaviors	[111]
Lipopolysaccharide-induced depressive-disorders-like behaviors in C57BL6/J mice	Sodium butyrate	Suppressing LPS-induced enhancement of pro-inflammatory cytokines like tumor necrosis factor-α, IL-1β, and IL-6 in the prefrontal cortex and hippocampus and inhibiting oxido-nitrosative stress	[112]
Induced depressive-disorders-like behaviors using chronic restraint stress in mice	Sodium butyrate	Normalizing acetylation of histone H3, HDAC2, and BDNF expression levels reduced by stress in the hippocampus	[113]
Chronic unpredictable mild stress (CUMS)-induced depressive-disorders-like behaviors in mice	Sodium butyrate	Restoring CUMS-induced BBB impairments by enhancing the expression of Occludin and ZO-1 proteins in the hippocampus, elevating serotonin concentration, and BDNF level	[114]
Forced swim and tail suspension induced immobility and depressive-disorders-like behaviors in rats	Sodium butyrate	Antidepressant-like effect of sodium butyrate through increases in histone H4 acetylation at the promoter of the transthyretin gene	[61]
Depressive- and anxiety-like behaviors induced by paclitaxel (PTX) in mice	Sodium butyrate	Alleviating depressive- and anxiety-like behaviors induced by PTX via restoring PTX-induced altered gut barrier integrity, microbiota composition, and food intake	[115]
Chronic restraint stress model in mice	Sodium phenyl butyrate	Reducing depressive-like behaviors by inhibiting oxido-nitrosative stress, neuro-inflammation, endoplasmic reticulum stress cascade, and restoring BDNF	[116]
CUMS in adult Sprague–Dawley rats	Sodium propionate	Inducing antidepressant effect by low- dose propionate (2 mg/kg/day)	[27]
CUMS in adult Sprague–Dawley rats	Sodium propionate	Inducing antidepressant effects via differential rescue of neurotransmitters in the prefrontal cortex after short-term intrarectal administration of propionate	[117]
Chronic social failure stress (CSDS) in mice	Acetate	Improving depressive-disorders-like behaviors by reducing the transcription of HDAC2, HDAC5, HDAC7, HDAC8, and increasing the transcription of HAT and P300, elevating the content of Ac-CoA in the nucleus, and subsequently promoting histone H3 and H4 acetylation	[118]
PCOS-associated depressive disorders in female Wistar rats	Acetate	Reducing the expression of HDAC2 and DNA methyltransferase in the prefrontal cortex and hippocampal tissues; inhibiting inflammation and oxidative stress; reducing depressive-like behaviors after acetate administration	[119]

## Data Availability

Not applicable.
